# *In silico* design of a multi-epitope vaccine against *Mycobacterium avium* subspecies *paratuberculosis*

**DOI:** 10.3389/fimmu.2025.1505313

**Published:** 2025-01-28

**Authors:** Weiqi Guo, Xinyu Wang, Jiangang Hu, Beibei Zhang, Luru Zhao, Guangdong Zhang, Jingjing Qi, Zuzhang Wei, Yanqing Bao, Mingxing Tian, Shaohui Wang

**Affiliations:** ^1^ Shanghai Veterinary Research Institute, Chinese Academy of Agricultural Sciences, Shanghai, China; ^2^ Laboratory of Animal Infectious Diseases and Molecular Immunology, College of Animal Science and Technology, Guangxi University, Nanning, China

**Keywords:** multi-epitope vaccine, *Mycobacterium avium* subspecies *paratuberculosis*, pan-genome, reverse vaccinology, molecular dynamics

## Abstract

The widespread chronic enteritis known as Paratuberculosis (PTB) or Johne's disease (JD) is caused by *Mycobacterium avium* subspecies *paratuberculosis* (MAP), posing a significant threat to global public health. Given the challenges associated with PTB or JD, the development and application of vaccines are potentially important for disease control. The aim of this study was to design a multi-epitope vaccine against MAP. A total of 198 MAP genomes were analyzed using pan-genome and reverse vaccinology approaches. B-cell and T-cell epitope analysis was performed on the selected promising cross-protective antigens followed by selection of epitopes with high antigenicity, no allergenicity, and no toxicity for the design of the vaccine. The designed vaccine was evaluated through molecular dynamics simulations, molecular docking, and immunological simulations. The results revealed the identification of five promising cross-protective antigens. In total, 10 B-cell epitopes, 10 HTL epitopes, and 9 CTL epitopes were selected for the design of the vaccine. Both the vaccine candidate and the vaccine-TLR4 complex demonstrated considerable stability in molecular dynamics simulations. Molecular docking studies confirmed that the vaccine candidate successfully interacted with TLR4. Immunological simulations showed an increase in both B-cell and T-cell populations after vaccination. Additionally, the vaccine candidate exhibited a codon adaptability index of 1.0 and a GC content of 53.64%, indicating strong potential for successful expression in *Escherichia coli*. This research developed a multi-epitope vaccine targeting MAP through pan-genomes and reverse vaccinology methods, offering innovative strategies for creating effective vaccines against MAP.

## 1 Introduction

Paratuberculosis (PTB), also known as Johne’s disease, is a prevalent disease recognized chronic in ruminants, caused by *Mycobacterium avium* subspecies *paratuberculosis* (MAP). It significantly impacts the dairy industry, reducing milk yields, slaughter value, and fertility, while spreading infection and increasing disease susceptibility (1–5). An estimated 90% of dairy herds in the United States are affected by MAP infection, leading to an annual economic loss of about $250 million nationwide (6, 7). Diagnosing MAP infection is extremely difficult and often goes unnoticed due to its subtle nature. Furthermore, MAP infections raise considerable public health concerns as they may potentially contribute to human disorders such as Crohn’s disease and thyroid disorders (8–11). Individuals infected with MAP are capable of excreting viable bacteria in their feces and milk; therefore, utmost care must be taken during the milking process to prevent fecal contamination (12), which poses a risk to global public health and socioeconomic stability.

Currently, various vaccine development methods are under evaluation against MAP. These include whole-cell vaccines (13), inactivated and attenuated live vaccines (14–20), and subunit vaccines (21–25). Live attenuated vaccines may be shed into the environment and could potentially revert to a virulent form. For inactivated vaccines, antigens essential for protection may have been removed or altered in a way that they are no longer immunogenic (26, 27). Furthermore, whole-cell vaccines contain a range of proteins that are irrelevant for protection (28), and the cell wall of MAP may even have immune regulators that could interfere with the development of a protective immune response (29).Recently, multi-epitope vaccines have garnered significant attention due to their departure from conventional single-epitope vaccine design strategies. These vaccines confer notable advantages over traditional options. They are cost-effective, safe, highly specific, easily preserved, and capable of modulating immune response types (30–32). The intricate architecture of multi-epitope vaccine is carefully designed to elicit a robust and comprehensive immune response, integrating an array of epitopes selectively recognized by diverse major histocompatibility complex (MHC) types. Its formulation enables the specific engagement of varied T cell subsets, thereby enhancing its immunogenic potential. Within this immune orchestration, cytotoxic T-lymphocyte (CTL) epitopes, helper T-lymphocyte (HTL) epitopes, and B cell epitopes each occupy pivotal positions, each indispensable for distinct facets of long-lasting immunity and pathogen resistance (33, 34). Additionally, the addition of adjuvant components in the MEVs leads to enhanced immunogenicity and induces a sustained immune response, leading to long-lasting immunity (33, 35, 36). This strategic approach endeavors to elicit a potent and multifaceted immune response, providing a broader and more effective shield against various diseases.

The focus of this research lies in formulating a multi-epitope vaccine directed against MAP, with a key emphasis on incorporating numerous epitopes to provoke a broad-spectrum and potent immunological response to combat the pathogen. Utilizing pan-genomes and reverse vaccinology methodologies, a total of five potential cross-protective antigens were identified from 198 MAP genomes. Drawing upon the amino acid sequences of these proteins, we crafted a 594-residue multi-epitope vaccine, meticulously selecting 10 B-cell epitopes, 10 HTL epitopes, and 9 CTL epitopes. Complementary in silico assessments further revealed encouraging attributes of this vaccine. Notably, this study marks the first report on the design of a multi-epitope vaccine targeting MAP, leveraging pan-genomic analysis, thereby setting the stage for the advancement of efficacious vaccines against MAP.

## 2 Materials and methods

**Figure 1:**
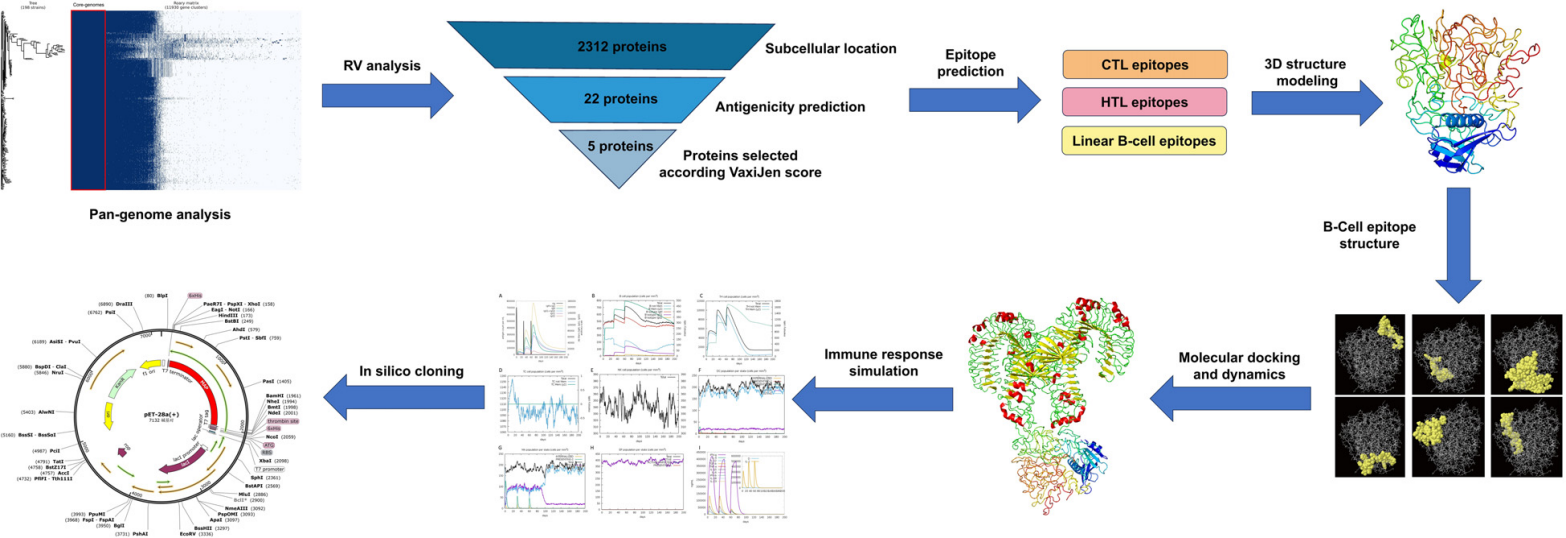
Stepwise methodology of the entire study. The workflow includes multiple phases: pan-genome and reverse vaccinology analyses, epitope prediction from the selected protein (CTL, HTL, and B-cell epitopes), vaccine construction and quality assessment, molecular docking with the TLR4 receptor, and molecular dynamics simulations (MDS) to evaluate the vaccine’s stability. Lastly, codon adaptation and immune simulation.

The step-by-step process used for this whole analysis is demonstrated in **Figure 1**.

### 2.1 Genome retrieval and pan-genome analysis

A comprehensive collection of 198 MAP isolate genomes was sourced from NCBI. The CDSs of all genomes underwent annotation with Prokka (version 1.14) (37). Utilizing Roary (version 3.13.0) (38), the proteomes of 198 samples were analyzed with the aim of determining the core proteomes; this was done using the default settings.

### 2.2 Prediction of subcellular localization and antigenicity

Following the identification of core proteomes through pan-genome analysis, subcellular localization was predicted using the PSORTb server (39). Subsequently, the antigenicity of the curated protein amino acid sequences was meticulously evaluated using VaxiJen (40).

### 2.3 Prediction of antigen epitope

To ensure the efficacy and safety of our multi-epitope vaccine, we leveraged various bioinformatic tools to thoroughly evaluate and refine our epitope selections. CTL epitopes were predicted using the NetCTL 1.2 server, which supports predictions for CTL epitopes restricted to 12 MHC class I supertypes: A1, A2, A3, A24, A26, B7, B8, B27, B39, B44, B58, and B62. MHC class I binding and proteasomal cleavage employ artificial neural networks, while TAP transport efficiency is forecasted using a weight matrix (41). Peptides were predicted to bind to MHC class II molecules using the IEDB MHC class II binding prediction tool, based on the NetMHCIIpan-4.1 method and including HLA class II alleles from three supertypes: HLA-DR, HLA-DQ, and HLA-DP. B-cell epitopes were identified using the ABCpred server (41–43). All predicted epitopes were then evaluated for allergenicity using AllergenFP and AllerTOP (44, 45), toxicity using ToxinPred, and antigenicity using VaxiJen (v2.0). This multi-layered screening process ensured that only non-toxic, non-allergenic, and antigenic epitopes were selected for inclusion in our vaccine design.

### 2.4 Design of multi-epitope vaccine

In the design of the multi-epitope vaccine, we integrated previously identified T-cell and B-cell epitopes. To facilitate efficient separation of these epitopes *in vivo* and circumvent the likelihood of unintended junctional epitope creation, tailored linkers were employed: AAY for CTL epitopes, GPGPG for HTL epitopes, and KK specifically for B-cell epitopes (46). In addition, the LTB subunit of the heat-stable enterotoxin from UniProt (Code: P0CK94) was used as an adjuvant. LTB is a potent mucosal adjuvant that induces both Th1 and Th2 immune responses, adaptable to different administration routes for tailored immunity. Its pentameric structure facilitates antigen delivery and enhances immune responses at both systemic and mucosal sites (47–50). This adjuvant was conjugated to the N-terminus of the vaccine via the EAAAK linker, Natural linkers like EAAAK are rigid and stable due to their tightly packed backbone and intrasegment hydrogen bonds, which promote α-helical structures, allowing them to act as rigid spacers between protein domains (51).

### 2.5 Prediction of allergenicity, antigenicity, and various physicochemical properties

The designed vaccine underwent further allergenicity evaluation using AllergenFP and AllerTOP (44, 45). The amino acid sequence composition of the multi-epitope vaccine underwent thorough antigenicity analysis utilizing the VaxiJen tool. Additionally, to gain a comprehensive understanding of its physicochemical attributes, the vaccine was evaluated with ProtParam. This analysis imparted crucial insights into the vaccine’s characteristics, including its stability, solubility, and potential immunogenicity (52).

### 2.6 Prediction of secondary structure

To delve deeper into the secondary structural components of our vaccine construct, we utilized the freely accessible PSIPRED server, an online protein structure analysis tool. By submitting the primary amino acid sequence of the vaccine peptide as input, PSIPRED predicted its secondary structure, offering invaluable insights into the folding patterns and conformational landscapes that underpin the vaccine’s structural integrity and potentially impact its functional performance (53).

### 2.7 Prediction, refinement, and quality assessment of the 3D structure of the developed multi-epitope vaccine

To guarantee heightened accuracy in forecasting the protein’s configuration, the initial three-dimensional blueprint, crafted by the Phyre 2 platform, underwent a meticulous optimization process facilitated by the GalaxyRefine system (54, 55). Following this refinement, the PDB documentation embodying the refined structure was subjected to a rigorous assessment of its tertiary structure’s integrity utilizing the SWISS-MODEL server as a tool (56). Additionally, the validity of the protein’s architecture was confirmed through the utilization of the ProSA web-based service (57).

### 2.8 Conformational prediction of the B-cell epitope

ElliPro (http://iedb.org/ellipro/) was used to identify B-cell epitopes within the protein’s refined 3D structure (58). It assigns a score, the Average Protrusion Index (PI), to each predicted epitope. Notably, the PI threshold for an ellipsoid that encompasses 90% of the protein residues is 0.9, while the remaining 10% lies outside this boundary. Determining this PI threshold relies on an assessment of the deviation from the center of mass for residues that lie outside the largest reasonable ellipsoid. In comparative evaluations, ElliPro has demonstrated superior accuracy in structure-based epitope prediction, evidenced by its Area Under the Curve (AUC)value of 0.732. This high AUC value underscores ElliPro’s reliability andprecision as a tool for predicting protein epitopes.

### 2.9 Molecular dynamics simulation of the multi-epitope vaccine

To mimic the intricate biological milieu encountered by proteins within living organisms, a molecular dynamics (MD) simulation was executed employing GROMACS (59). Furthermore, the durability and physical characteristics of the docked complex were meticulously evaluated through the iMODS web server (accessible at http://imods.Chaconlab.org) using normal mode analysis (NMA). The iMODS analysis divulged pivotal insights into the complex’s structural dynamics, offering a comprehensive assessment encompassing deformability, B-factor analysis, eigenvalue examination, variance measurements, covariance mapping and elastic network modeling.

### 2.10 Molecular docking of the multi-epitope vaccine structure with toll-like receptors

The designed vaccine was subjected to various docking simulations to evaluate its binding affinity towards human Toll-like receptors, including TLR4. The crystallized structures of TLR4, designated by the Protein Data Bank with the unique PDB code 3FXI. Molecular docking was conducted utilizing the web-based ClusPro tool (60). The ensuing three-dimensional representations of both the Toll-like receptors and the vaccine-TLR4 complexes were rendered visible using PyMOL software. The specific amino acid sequences that mediated the interplay between the tailored vaccine and TLR4 were pinpointed with the assistance of PDBsum, facilitating a comprehensive understanding of their interaction patterns (61).

### 2.11 Immune simulation analysis

To evaluate the immunological response elicited by the vaccine, we used C-ImmSim, an agent-based simulator that employs the Celada-Seiden model to simulate the mammalian immune response to the designed vaccine (62). This simulation generates profiles for both humoral and cellular immune responses. The default settings were retained, with modifications made to the “Simulation Steps” set at 600. Additionally, the timing of injections was adjusted, with the first injection administered on day 1, the second on day 30 and the third on day 60.

### 2.12 Codon adaptation and *in silico* cloning

The *Escherichia coli* K-12 strain was selected as the host for the in silico cloning process to create the vaccine construct. The codon sequence of the vaccine was optimized using the JCAT codon adaptation tool (63). Subsequently, the sequence was cloned into the pET28a(+) vector, leveraging the capabilities of SnapGene software for seamless insertion (64).

## 3 Results

### 3.1 Pan-genome analysis of MAP complete genomes

**Figure 2:**
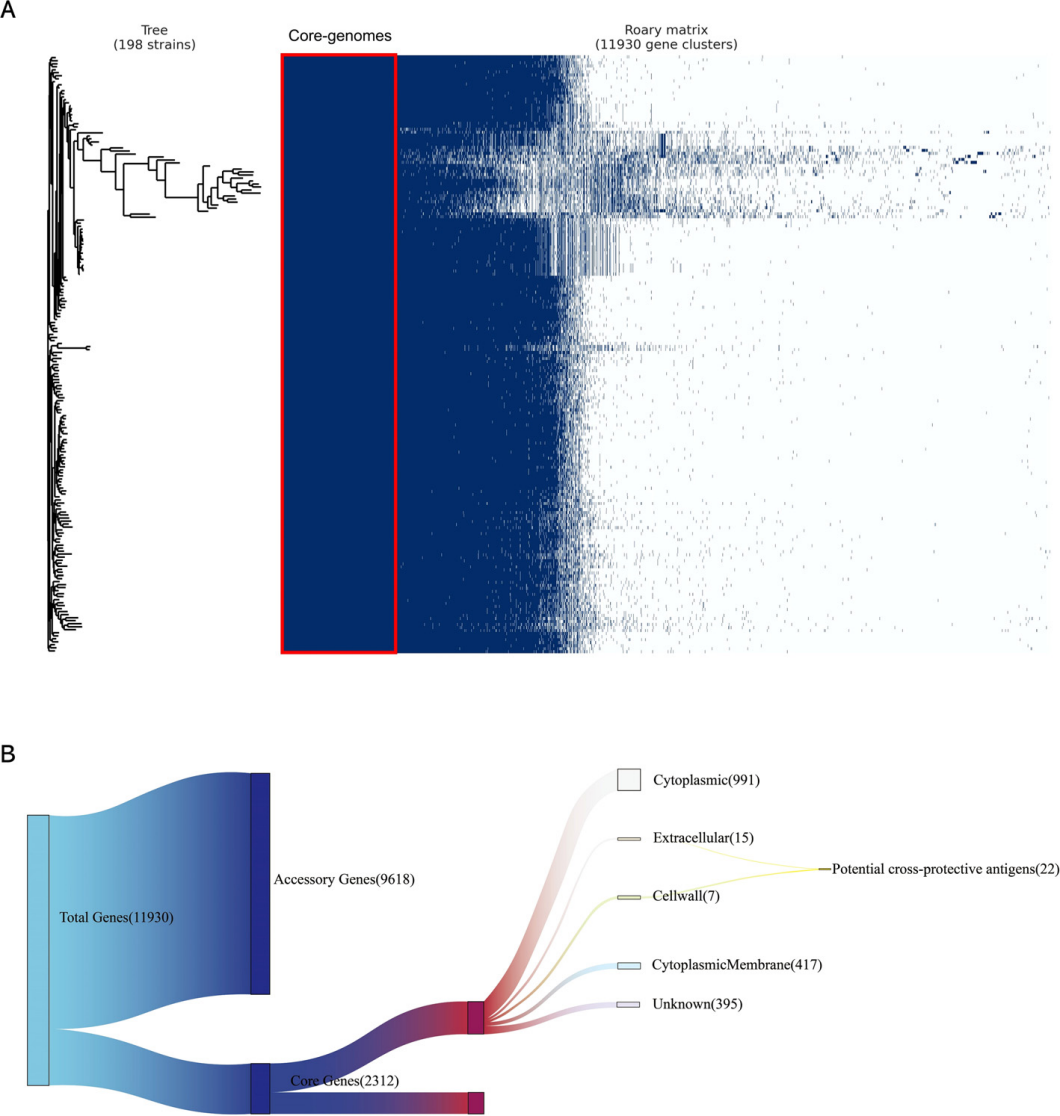
Pan-genome and reverse vaccinology analysis. **(A)** A whole-genome phylogenetic tree and a matrix indicating gene presence and absence were generated for 198 MAP genomes isolates. The left tree is a phylogenetic tree constructed based on coregenome alignment to show the evolutionary relationships among all MAP genomes used in this study; the right matrix is constructed based on the presence and absence matrix to show the clustering of genes in all MAP genomes used in this study, where the red boxes indicate the core-genomes used in this study. **(B)** Subtractive proteomics of core-proteome. Twenty-Two potential cross-protective antigens were identified in 2312 core proteins by subcellular location.

The annotation of 198 complete MAP genomes was displayed using Prokka, followed by the conversion of genomes into GFF3 format files. Subsequently, a pan-genome analysis was conducted utilizing Roary, which revealed a total of 11,930 genes among the 198 genomes. Among the identified genes, 2,312 were categorized as essential core genes. Our comprehensive analysis generated a whole-genome phylogenetic tree, along with a matrix that clearly indicates the distribution of both core and accessory genes across samples. This information is graphically shown in **Figure 2A**.

### 3.2 RV analysis for protein prioritization

A subcellular localization screening, utilizing PSORTb, classified 991 proteins as cytoplasmic, 417 proteins as cytoplasmic membrane-bound, 15 proteins as extracellular, 7 proteins as cell wall-associated, and 395 proteins had an unknown localization. Proteins with extracellular secretion and those in the cell wall were chosen for detailed analysis (**Figure 2B**).

The proteins identified in the preliminary stage underwent scoring by VaxiJen. From among these proteins, 22 were recognized as potential antigens, satisfying the default threshold of 0.4. Upon further screening, 5 proteins (**Table 1**) emerged with scores exceeding 0.7. These proteins were identified as high-priority candidates and were selected for epitope extraction to construct the multi-epitope MAP vaccine.

**Table 1:** Description of 5 potential protective antigens with VaxiJen score of > 0.7.

Protein	No. of amino acids	Subcellular location	VaxiJen score
group 6283	456	Extracellular	1.1373
LpqH	522	Extracellular	1.0029
group 7515	468	Extracellular	0.8778
group 7334	1056	Extracellular	0.7445
FbpA	1341	Extracellular	0.7022

### 3.3 Identification of CTL epitopes

Employing the updated NetCTL-1.2 platform, we forecasted CTL epitopes from the selected protein repertoire. Out of the 227 predicted epitopes, a subset of 9 were meticulously chosen, guided by their high binding affinity ratings with MHC I supertypes, alongside comprehensive evaluations of toxicity, allergen potential, and antigenicity. These non-toxic, non-allergenic, and antigenic epitopes, shown in **Table 2**, were deemed suitable for inclusion in the vaccine construct.

**Table 2:** Predicted CTL epitopes extracted from conserved potential antigens for the construction of multi-epitope vaccine.

Protein	Supertype	Residue no	Peptide sequence	VaxiJen	Allergen FP	AllerTOP	Toxinpred
group 6283	B39/B58/B62	78	GSMGTTTTL	1.1877	Non-Allergen	Non-Allergen	Non-Toxin
	B62	16	LTVAPAAAL	0.8849	Non-Allergen	Non-Allergen	Non-Toxin
LpqH	A3	12	ASAGCSSNK	1.7667	Non-Allergen	Non-Allergen	Non-Toxin
	B58	111	GAAVNGKTW	1.3383	Non-Allergen	Non-Allergen	Non-Toxin
group 7515	B7	55	APSESTGTS	1.9109	Non-Allergen	Non-Allergen	Non-Toxin
	B7	178	KPGDAEATV	1.6934	Non-Allergen	Non-Allergen	Non-Toxin
group 7334	B7/B44	104	RDTQGGSSL	2.4335	Non-Allergen	Non-Allergen	Non-Toxin
FbpA	B58	73	GANSPALYL	1.2268	Non-Allergen	Non-Allergen	Non-Toxin
	B62	36	GGSATAGAF	1.519	Non-Allergen	Non-Allergen	Non-Toxin

### 3.4 Identification of HTL epitopes

Using the IEDB server, we predicted HTL epitopes for three HLA supertypes: HLA-DR, HLA-DQ, and HLA-DP. We screened 10 non-toxic, non-allergenic, and antigenic epitopes for inclusion in the vaccine construct, as shown in **Table 3**.

**Table 3:** Predicted HTL epitopes extracted from conserved potential antigens for the construction of multi-epitope vaccine.

Protein	Allele	Start	End	Peptide	Vaxijen	Allergen FP	AllerTOP	Toxinpred
group 6283	HLA-DRB1*01:01	9	23	VRDRWLRLTVAPAAA	0.7388	Non-Allergen	Non-Allergen	Non-Toxin
	HLA-DRB1*09:01	117	131	TNNKITFDKVNTRIT	0.8423	Non-Allergen	Non-Allergen	Non-Toxin
LpqH	HLA-DQA1*05:01/DQB1*03:01	21	35	SNTGASGSSGAPAAA	1.6873	Non-Allergen	Non-Allergen	Non-Toxin
	HLA-DRB1*09:01	107	121	GGNAGAAVNGKTWAI	1.5948	Non-Allergen	Non-Allergen	Non-Toxin
group 7515	HLA-DQA1*04:01/DQB1*04:02	174	188	TITWKPGDAEATVTT	1.4843	Non-Allergen	Non-Allergen	Non-Toxin
	HLA-DQA1*05:01/DQB1*02:01	16	30	AAFALIGGACSKSNN	1.2097	Non-Allergen	Non-Allergen	Non-Toxin
group 7334	HLA-DQA1*03:01/DQB1*03:02	34	48	KSGTTIHVTEYSTAT	1.0325	Non-Allergen	Non-Allergen	Non-Toxin
	HLA-DRB1*09:01	8	22	LALTACLTAPGATAD	0.992	Non-Allergen	Non-Allergen	Non-Toxin
FbpA	HLA-DQA1*03:01/DQB1*03:02	277	291	RTSNLKFQDAYNGAG	1.5673	Non-Allergen	Non-Allergen	Non-Toxin
	HLA-DRB1*09:01	53	67	YLQVPSAAMGRDIKV	1.275	Non-Allergen	Non-Allergen	Non-Toxin

### 3.5 Identification of B-cell epitopes

To predict B-cell epitopes, the ABCpred server was utilized with an epitope identification threshold of 0.5. Among the 102 epitopes screened based on their amino acid sequence positions, 10 non-toxic, non-allergenic, and antigenic epitopes were selected for inclusion in the final vaccine construct., as shown in **Table 4**.

**Table 4:** Predicted B-cell epitopes extracted from conserved potential antigens for the construction of multi-epitope vaccine.

Protein	Start position	Sequence	Score	VaxiJen	Allergen FP	AllerTOP	Toxinpred
group 6283	50	TSAGAAPTTGSGGAST	0.94	2.106	Non-Allergen	Non-Allergen	Non-Toxin
	114	VSGTNNKITFDKVNTR	0.89	1.316	Non-Allergen	Non-Allergen	Non-Toxin
LpqH	62	TIGIGDPTAGLGAVVS	0.88	0.835	Non-Allergen	Non-Allergen	Non-Toxin
	37	PQLIVDGQTQNVSGQV	0.85	1.345	Non-Allergen	Non-Allergen	Non-Toxin
group 7515	54	AAPSESTGTSGAPSST	0.8	1.553	Non-Allergen	Non-Allergen	Non-Toxin
	35	TSSASSSATSSATSGT	0.73	1.905	Non-Allergen	Non-Allergen	Non-Toxin
group 7334	55	SATWVSSGCPGGGGCN	0.9	1.769	Non-Allergen	Non-Allergen	Non-Toxin
	61	SGCPGGGGCNVIELTI	0.89	2.125	Non-Allergen	Non-Allergen	Non-Toxin
FbpA	92	NGWDINTPAFEWYNQS	0.77	1.223	Non-Allergen	Non-Allergen	Non-Toxin
	62	GRDIKVQFQSGGANSP	0.67	1.624	Non-Allergen	Non-Allergen	Non-Toxin

### 3.6 Multi-epitope vaccine construction

**Figure 3:**
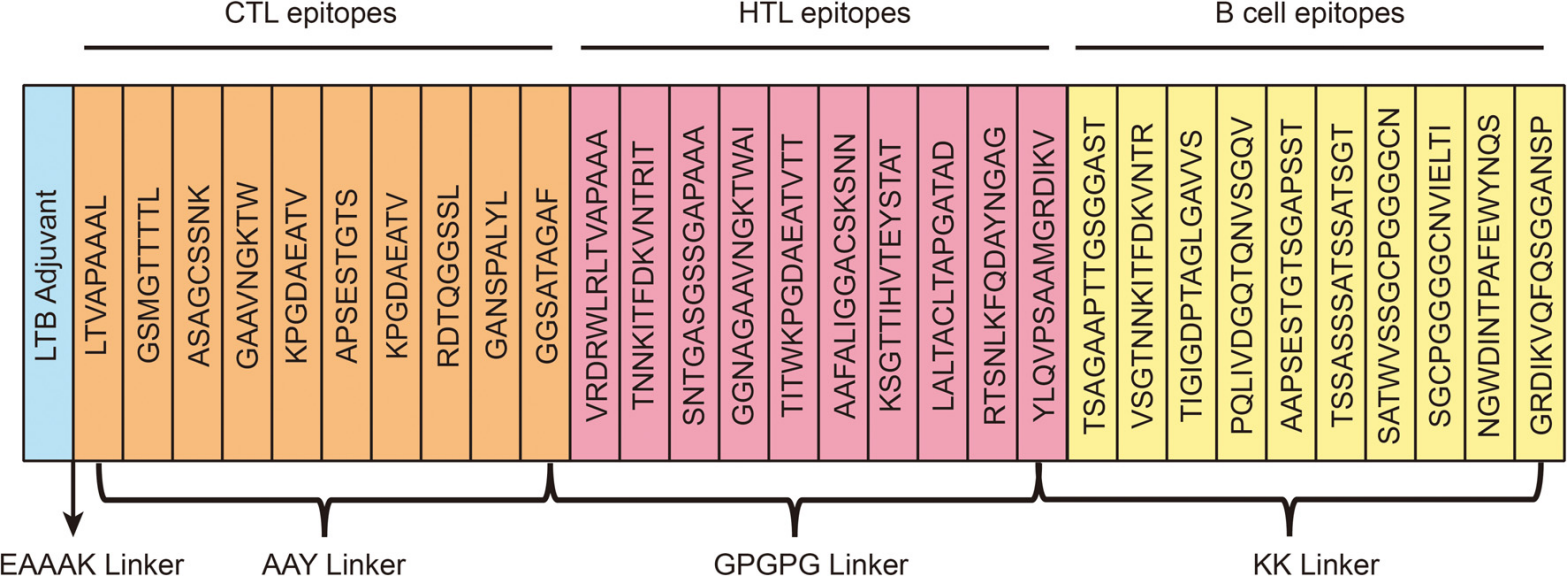
A schematic illustration of the final multi-epitope vaccination development. The multi-epitope vaccine possesses 594 amino acids including adjuvant (light blue color), epitopes (CTL-orange, HTL-pink, B-cell-yellow), and linkers (EAAAK, AYY, KK, GPGPG).

Using established criteria, the vaccine construct was carefully designed by integrating selected epitopes. A total of 10 B-cell epitopes, 10 HTL epitopes, and 9 CTL epitopes were screened. The B-cell epitopes were linked using the KK linker, HTL epitopes with GPGPG, and CTL epitopes with AAY. The LTB adjuvant was conjugated to the N-terminus of the vaccine via the EAAAK linker. The final vaccine construct, consisting of 594 amino acids. A graphical representation of the vaccine is shown in **Figure 3**.

### 3.7 Immunological and physiochemical properties

The vaccine’s potential for allergenicity was assessed by analysis on both the AllergenFP and AllerTOP servers. Both platforms indicated that the vaccine sequence is likely a nonallergenic. To further confirm its antigenicity, the VaxiJen server was used for reassessment, which produced a score of 1.1949.

The web server ProtPara was utilized to evaluate various physicochemical properties of our designed vaccine. Comprising 594 amino acids, includes 33 negatively charged and 56 positively charged residues, with a molecular weight of 58 kDa. The theoretical PI of the vaccine was predicted to be 9.52, containing 8205 atoms, and the chemical representation was C_2551_H_4066_N_732_O_841_S_15_. The predicted half-life of the vaccine is 30 hours *in vitro*, more than 20 hours in yeast (*in vivo*), and over 10 hours in *Escherichia coli* (*in vivo*). The instability index of the protein was 27.26, which is considered stable. Notably, its aliphatic index of 58.69 suggests relatively higher thermostability. Additionally, the vaccine demonstrates a hydrophilic nature, with a grand average of hydropathicity (GRAVY) value of -0.341.

### 3.8 Prediction of secondary structure

**Figure 4:**
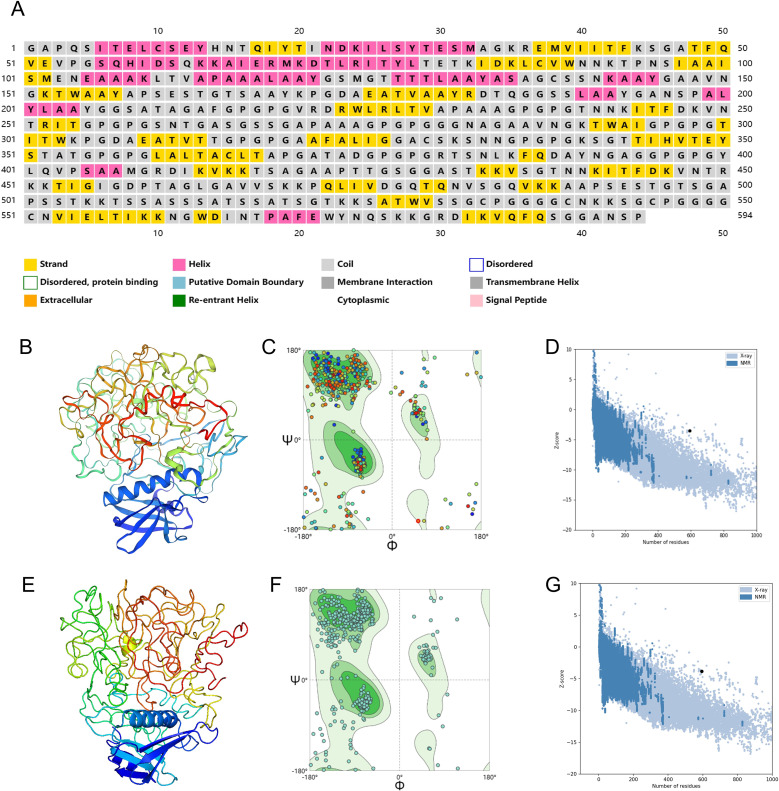
Secondary and three-dimensional structure prediction and quality assessment. **(A)** Secondary structure prediction to vaccine sequence by PSIPRED server. **(B)** The 3D structure of the designed initial vaccine predicted using the Phyre 2 server. **(C)** Ramachandran plot of the initial structure of the designed vaccine. **(D)** Z-score plot for the 3D structure of the initial vaccine. **(E)** The 3D structure of the designed final vaccine predicted using the Phyre 2 server. **(F)** Ramachandran plot of the final structure of the designed vaccine. **(G)** Z-score plot for the 3D structure of the final vaccine.

The PSIPRED server was employed to predict the secondary structure of the chimeric peptide, which consists of 594 amino acid residues. The prediction revealed a composition comprising 14.14% alpha helix, 23.23% beta-sheet, and a noteworthy 62.63% of coils, shown in **Figure 4A**. This breakdown hints at a preponderance of coils in the vaccine’s structure, with smaller proportions of alpha helices and beta-sheets. The arrangement of these secondary structures is crucial for determining the vaccine’s stability, solubility, and immunogenic potential.

### 3.9 Tertiary structure, refinement and validation

To refine the three-dimensional (3D) structure of the designed vaccine, the construction process first took place on the Phyre 2 platform, Visualization of·initial vaccine model was achieved through PyMOL, as shown in **Figure 4B**. An optimal model typically boasts a higher concentration of residues located in the Ramachandran-favored region, along with a lower proportion in the outlier and rotamer regions. A detailed analysis of the Ramachandran plot for the initial tertiary structure revealed that 78.38% of residues fell within the favored region, 7.43% were outliers, and 0% belonged to the rotamer region (**Figure 4C**). The MolProbity and Clash scores for this structure were 2.85 and 41.1, respectively. Additionally, the ProSA tool was used to evaluate the quality of the initial structure, yielding a Z-score of -3.51, as shown in **Figure 4D**.

The vaccine’s initial three-dimensional structure was refined using the GalaxyRefine server. Among the 5 generated models, the one boasting a Rama favored score of 81.1 was chosen for deeper analysis. Visualization of·the selected model was achieved through PyMOL, as shown in **Figure 4E**, the vaccine’s finalized structure exhibited improved metrics, with 80.24% of residues in the Ramachandran-favored region, 5.57% as outliers, and 0.95% in the rotamer region (**Figure 4F**). The MolProbity and Clash scores improved to 1.96 and 4.50, respectively. The ProSA Z-score also improved to -3.83, further confirming that the refined structure aligns even more closely with experimentally determined structures (**Figure 4G**).

### 3.10 Prediction of B-cell epitope structure

**Figure 5:**
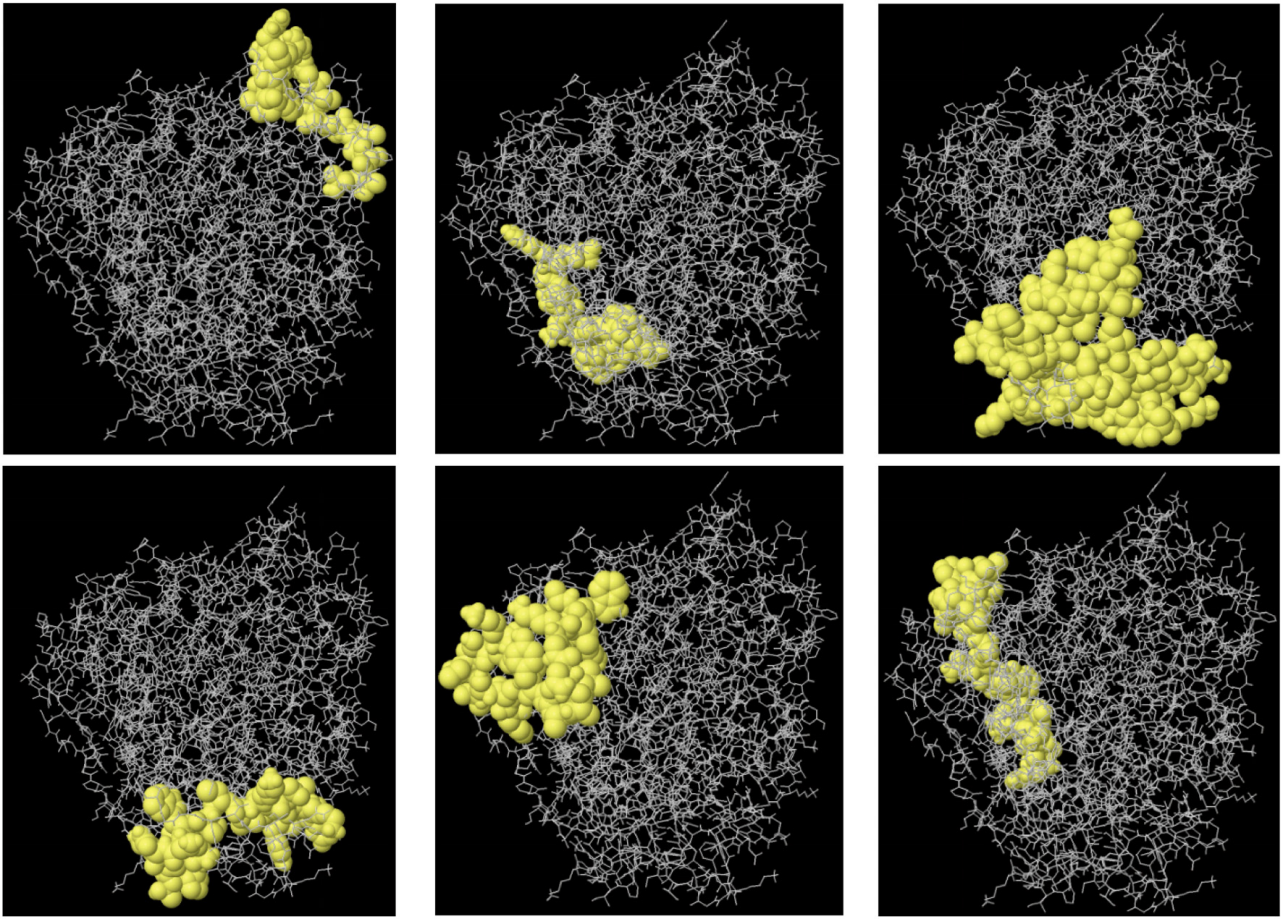
The 3D structure B-cell epitopes of the designed vaccine were prediction using the ElliPro tool. Sticks in gray represent the vaccine component, while surfaces in yellow represent conformational B cell epitopes.

The ElliPro tool was used to predict the conformational B-cell epitopes in the multi-epitope vaccine. Epitopes with a score of 0.7 or above were chosen for further evaluation. This selection revealed five discontinuous B-cell epitopes, comprising approximately 270 residues with scores ranging from 0.722 to 0.793. The number of residues within each conformational epitope varied between 20 and 52 (**Figure 5**).

### 3.11 Molecular docking exploration

**Figure 6:**
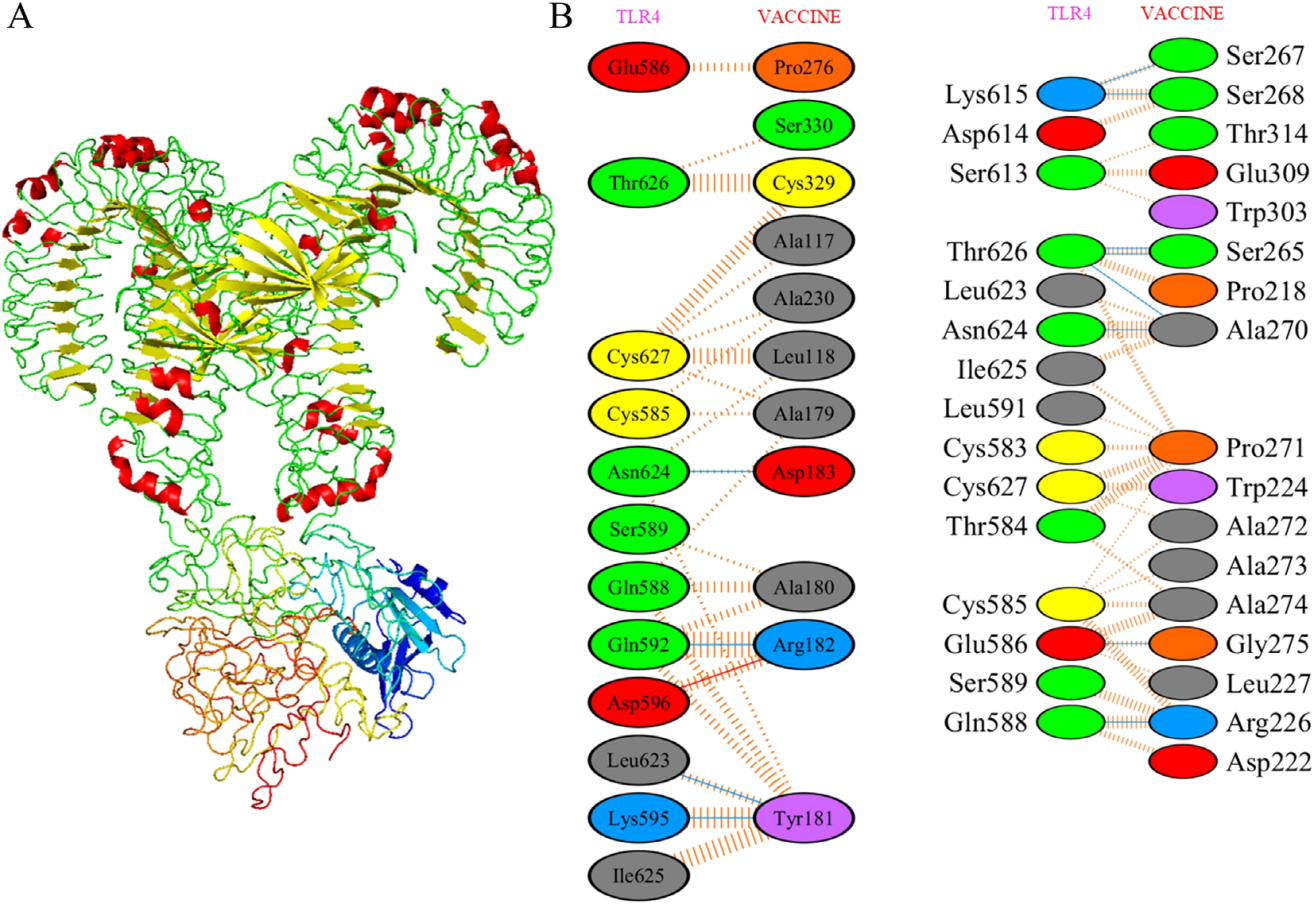
Molecular docking of designed vaccine with Toll-like receptors. **(A)** Vaccine-TLR4 docked complex. **(B)** Residue interaction between designed vaccine and TLR4. Molecular interaction study of docked complex by PDBsum where red for salt bridges, yellow for disulfidebond, blue for hydrogen bond and orange for non-bonded contacts.

Molecular docking simulations between the designed vaccine and TLR4 were performed using the ClusPro server. Each simulation produced 30 clusters, and the cluster with the most favorable energy score of -1037.4 was selected as the best docking arrangement. To visualize these docking outcomes, we employed PyMOL (**Figure 6A**).

To achieve a deeper comprehension of the vaccine’s interplay with TLR, we analyzed the participating residues and the binding strength of the receptor-ligand interaction through the PDBsum tool. In the vaccine-TLR4 docking complex (**Figure 6B**), the vaccine presented 33 interface residues, while TLR4 contributed 31, with respective interface areas of 1612 Å2 and 1562 Å2. This complex exhibited 2 salt bridges, 18 hydrogen bonds and 183 non-bonded contacts, providing strong evidence of the strong affinity between the vaccine and TLR4.

### 3.12 Molecular dynamics simulations of the vaccine and Vaccine-TLR4 complex

Molecular dynamics simulations were performed using GROMACS to assess the biological stability of the vaccine and the vaccine-TLR4 complex. For both simulations, the OPLS-AA force field was employed, with the vaccine centrally positioned in a water-filled cubic box and chloride ions added to maintain neutrality.

**Figure 7:**
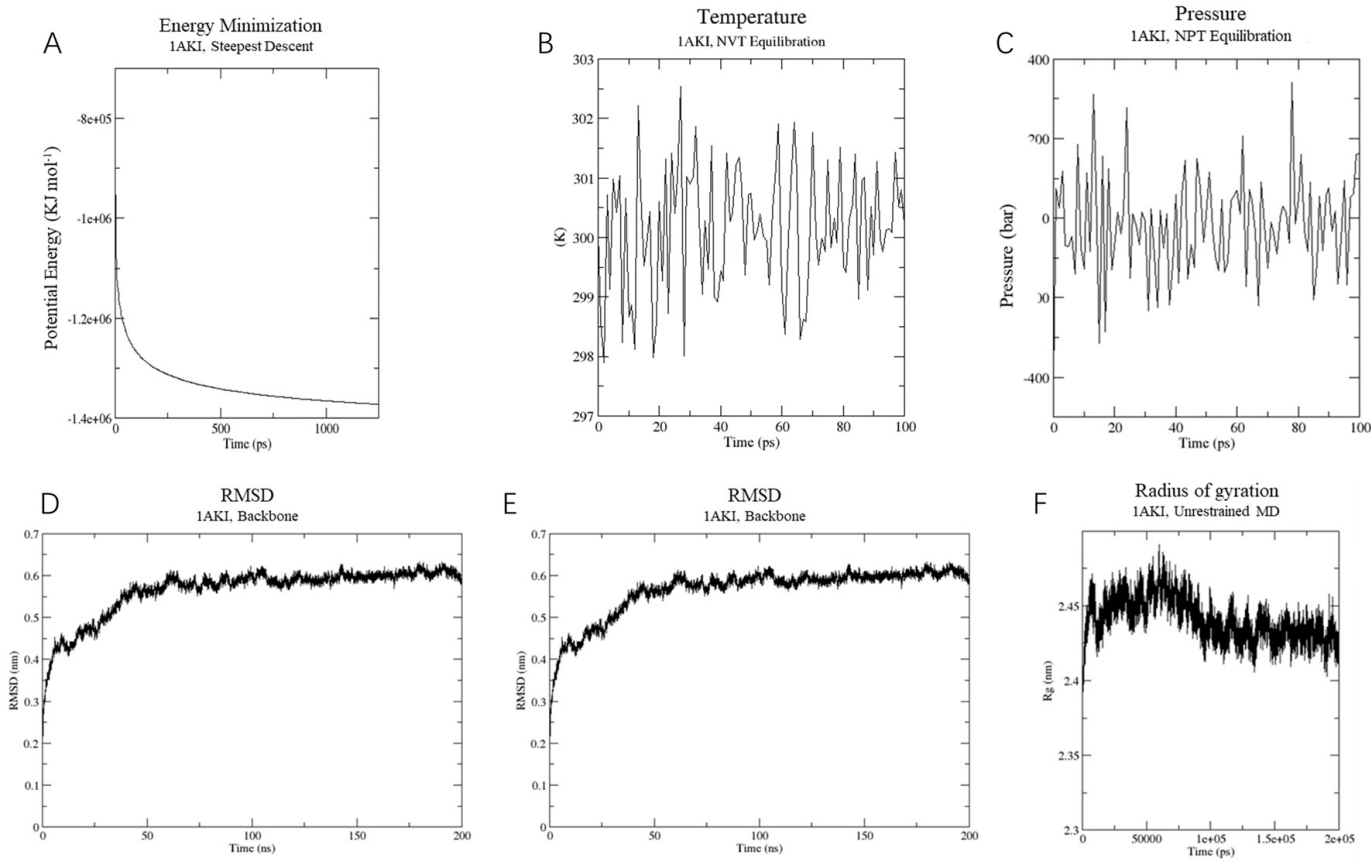
Molecular dynamics simulation of vaccine construct. **(A)** The process of system energy minimization. **(B)** Temperature (Kelvin) fluctuation of the ligand-receptor complex during the equilibration phase (100 ps). **(C)** The ligand-receptor pressure plot, measured over the equilibration phase (100 ps). **(D)** Root mean square deviation (RMSD) plot reflect the stability of the designed vaccine. **(E)** Illustrates the RMSF for the final vaccine construct. **(F)** Rg analysis of vaccine construct during a 200 ns simulation analysis.

In the vaccine protein simulation, energy minimization reduced the system’s energy to approximately -14,000,000 KJ/mol (**Figure 7A**), followed by equilibration at 300 K and 1 atm using NVT and NPT ensembles (**Figures 7B, C**). RMSD analysis revealed a stable conformation with an RMSD of about 0.6 nm after 50 ns (**Figure 7D**). RMSF results indicated high stability, with an average RMSF of 0.45 ± 0.3 Å and flexible residues fluctuating around 0.8 Å (**Figure 7E**). The Rg analysis showed a compact and stable structure, with an average Rg of 2.45 ± 0.05 Å (**Figure 7F**). These findings suggest that the vaccine protein is stable and suitable for further experimental validation.

**Figure 8:**
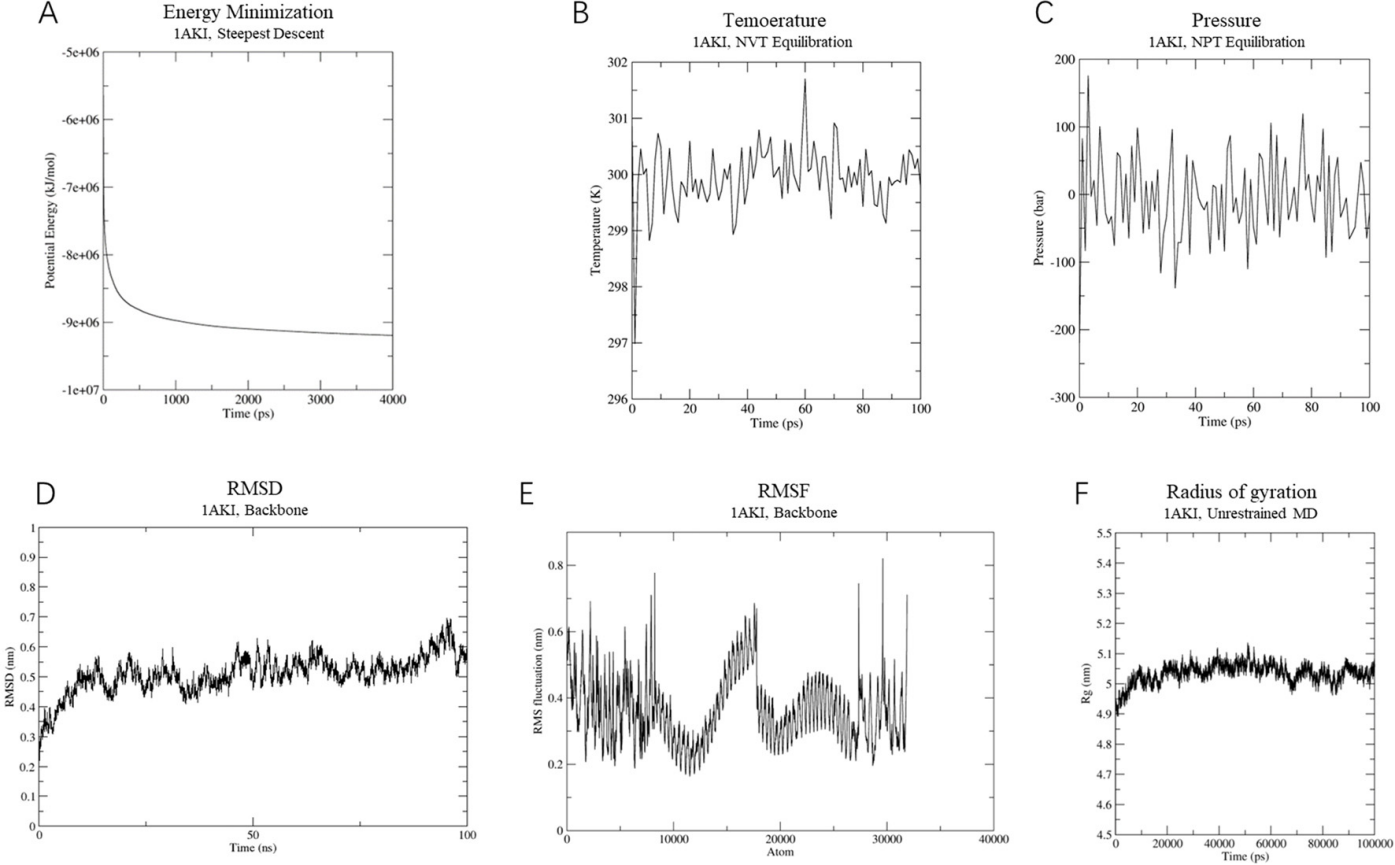
Molecular dynamics simulation of vaccine-TLR-4 complex. **(A)** The process of system energy minimization. **(B)** Temperature (Kelvin) fluctuation of the ligand-receptor complex during the equilibration phase (100 ps). **(C)** The ligand-receptor pressure plot, measured over the equilibration phase (100 ps). **(D)** Root mean square deviation (RMSD) plot reflect the stability of the designed vaccine. **(E)** Illustrates the RMSF for the final vaccine-TLR4 complex. **(F)** Rg analysis of MEVC-TLR-4 during a 100 ns simulation analysis.

In the vaccine-TLR4 complex simulation, energy minimization lowered the system’s energy to around -9,000,000 KJ/mol (**Figure 8A**). The system was similarly equilibrated at 300 K and 1 atm using NVT and NPT ensembles (**Figures 8B, C**). RMSD analysis showed a consistent conformation with an RMSD of approximately 0.5 nm after 15 ns (**Figure 8D**). The RMSF results averaged 0.5 ± 0.3 Å, with flexible residues fluctuating around 0.8 Å (**Figure 8E**). The Rg analysis indicated a compact and stable structure, with an average Rg of 5 ± 0.1 Å (**Figure 8F**). Overall, the vaccine-TLR4 complex maintained a consistent and stable structure, indicating high stability throughout the simulation.

**Figure 9:**
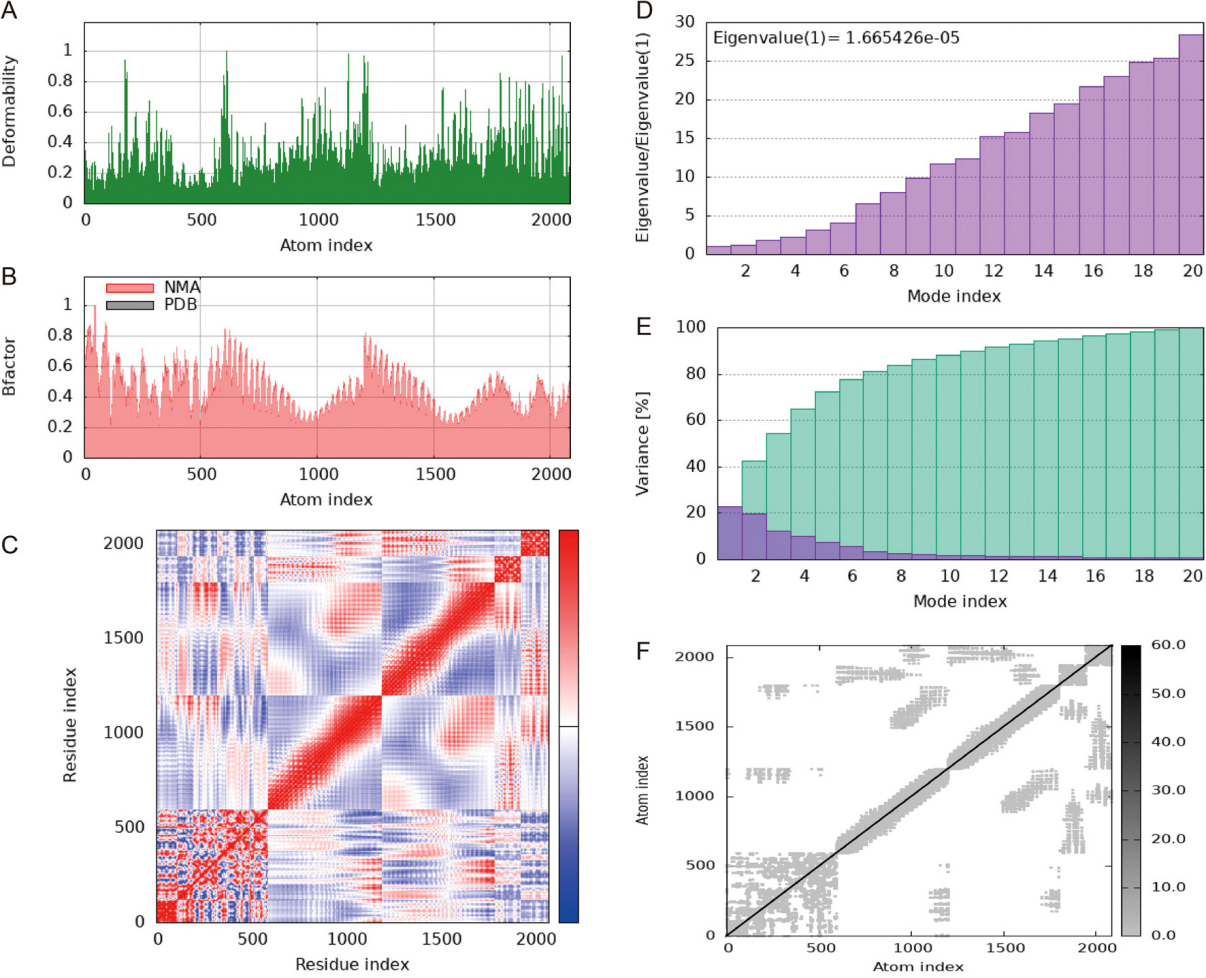
Molecular dynamics simulation of a vaccine-TLR4 includes several aspects. **(A)** Analysis of deformability through molecular dynamics simulations. **(B)** Examination of B-factors. **(C)** Covariance mapping, with red representing correlated regions, white demonstrating no correlation, and blue representing anti-correlation. **(D)** Evaluation of eigenvalues, where lower numbers indicate more facile deformation. **(E)** Analysis of variance, with red indicating individual variations and green indicating aggregate variances. **(F)** Elastic network analysis, where darker areas suggest increased stiffness.

Normal mode analysis (NMA) was conducted using the iMOD server to evaluate the stability of the vaccine within the vaccine-TLR4 complex. Regions containing hinge points exhibited considerable deformability, as depicted in **Figure 9A**. The B-factor values showed a direct correlation with the root mean square in the NMA (**Figures 9B, C**). The eigenvalues for the vaccine-TLR4 complex were calculated to be 1.665426 × 10^5^, indicating the energy required for structural deformation, where lower values suggest easier deformation (**Figure 9D**). The relationships between pairs of residues is visualized in a covariance matrix (**Figure 9E**), while the elastic network model depicted interatomic connectivity through springs (**Figure 9F**). These results suggest that the vaccine maintains a continuous interactions with TLR4.

### 3.14 Immune response simulation

**Figure 10:**
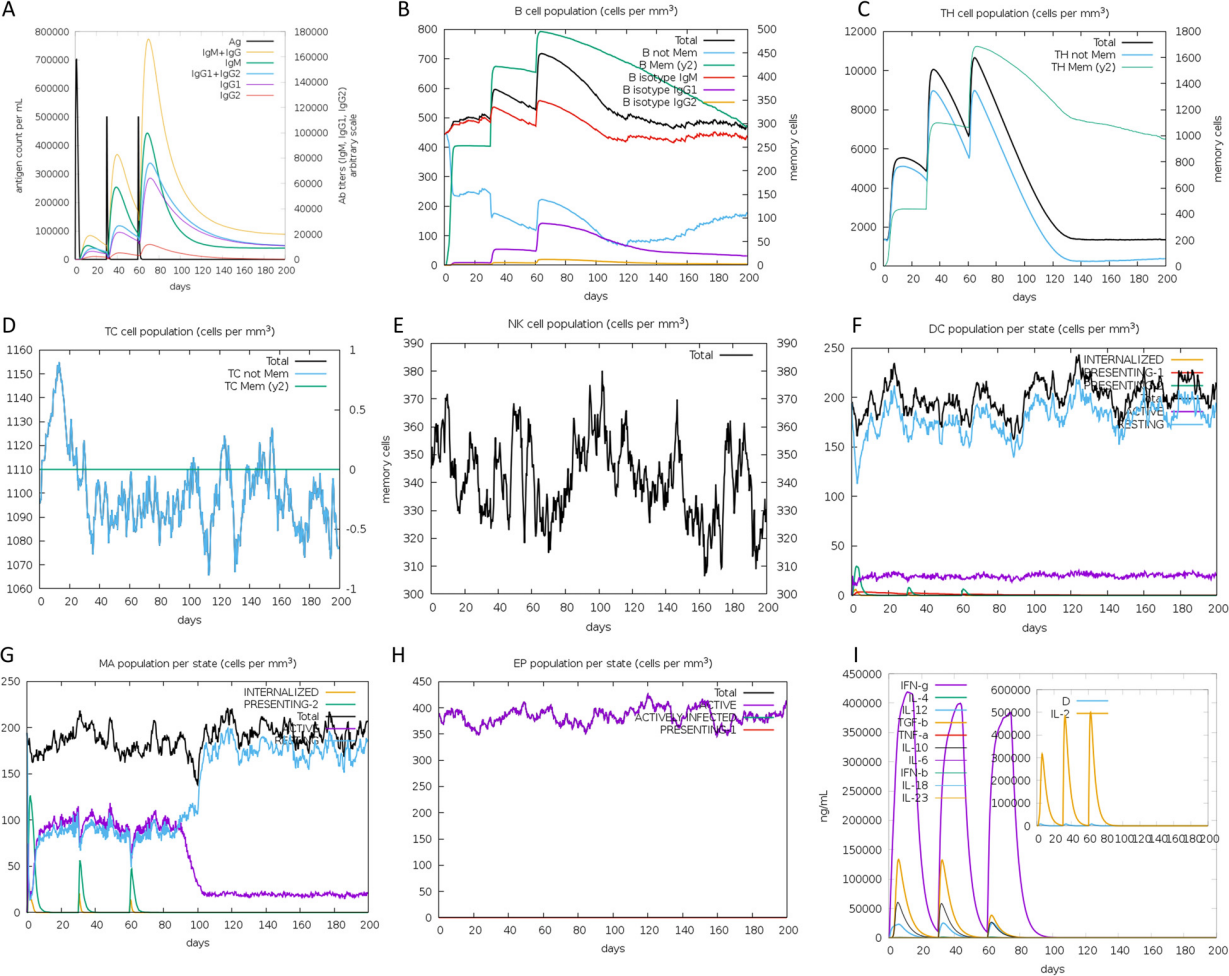
Immune simulation in silico. **(A)** Antigen and antibody levels in response to vaccine injection. **(B)** B-lymphocyte population after three injections of the vaccine. **(C)** B-lymphocyte population at each stage. **(D)** T-lymphocyte population after two injections of the vaccine. **(E)** T-lymphocyte population at each stage. **(F)** The population of CD8 T-cytotoxic lymphocytes at each state. **(G)** The population of dendritic cells at each state. The bars in the legend indicate “INTERNALIZED”, “PRESENTING-1”, “PRESENTING-2”, “Total”, “ACTIVE” and “RESTING”, respectively. **(H)** Macrophage population at each state. The bars in the legend indicate “INTERNALIZED”, “PRESENTING-2”, “Total”, “ACTIVE”, and “RESTING”, respectively. **(I)** Concentration of cytokines and interleukins, “D” in the inset plot indicates the danger signal.

The analysis of simulation results revealed several noteworthy findings. Firstly, there was a significant increase in antibody titers following subsequent immunizations, indicating a strong humoral immune response (**Figure 10A**). Secondly, the secondary immune response showed marked augmentation compared to the primary response, evident in the heightened activity of B cells (**Figures 10B, C**), helper T cells (**Figures 10D, E**), and cytotoxic T cells (**Figure 10F**). Additionally, the rise in dendritic cells (**Figure 10G**) and macrophage (**Figure 10H**) concentrations pointed to efficient antigen presentation by these APCs. Finally, cytokine and interleukin levels (**Figure 10I**) saw a notable surge following the second inoculation. In summary, these results suggest that the vaccine can stimulate high levels of antibodies, activated immune cells, cytokines, and APCs, underscoring its immunogenicity and potential efficacy against the targeted pathogen.

### 3.15 Codon adaption and *in silico* cloning

**Figure 11:**
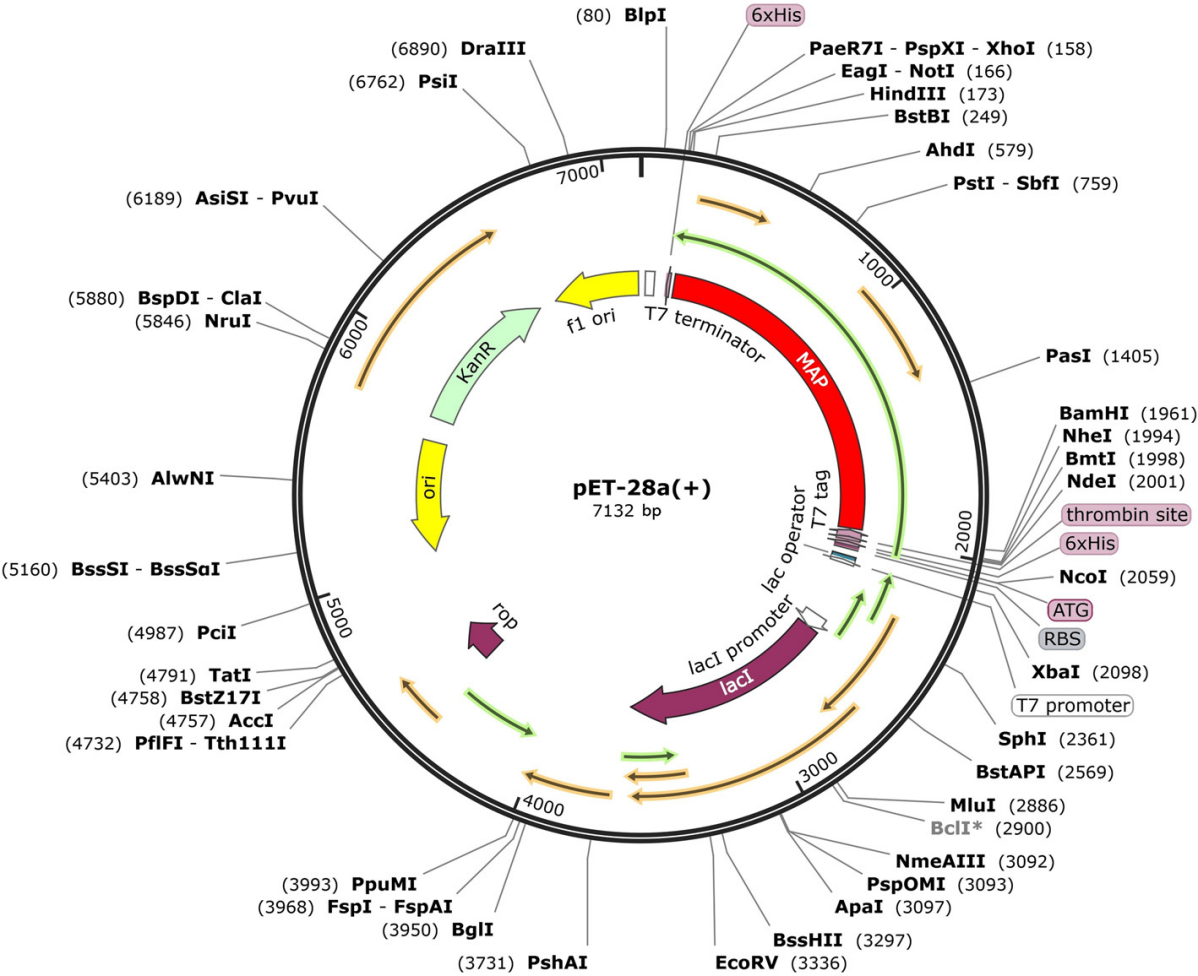
Expression of designed multiepitope *Mycobacterium avium* subspecies *paratuberculosis* vaccine constructed using pET28a (+). The designed multi-epitope vaccine is marked with red color, represents the pET28a (+) expression vector.

The JCat server facilitated the codon optimization of the vaccine’s DNA sequence, resulting in a CAI value of 1.0 and a GC content of 53.64%, thus ensuring high expression efficiency. With the aid of SnapGene software, the vaccine sequence’s successful insertion between the *BamH* I and *Hind* III restriction sites was visualized, appearing highlighted in gray within the constructed vector (**Figure 11**).

## 4 Discussion

Paratuberculosis (PTB) is a chronic and debilitating disease that significantly affects livestock production, leading to economic losses for marginal farmers (65). The disease causes herd losses due to high mortality rates, increased culling, reduced reproductive efficiency, reduced growth rates, and lower milk yields (66). The ability of MAP to persist in harsh conditions further complicates disease management and raises public health concerns (67–69). While vaccination has been shown to reduce MAP fecal shedding, clinical symptoms, and lesions in target organs of affected animals, it does not completely eliminate the pathogen or its effects (70–72).

Immuno-informatics, through its modern advancements, strengthens the pursuit of dependable, cost-efficient, effective, and safe vaccine candidates to combat infectious diseases. Subunit vaccinations, encompassing specific protein portions and potent adjuvants, serve as effective triggers for the immune system, conferring immunity to the host. Presently, the focus lies on subunit-based vaccines to design potential defenses against pathogenic agents. Numerous studies employing immuno-informatics have explored multi-epitope vaccines targeting various pathogens like *B Streptococcus* (73), *Mycobacterium Tuberculosis* (74), *Mycoplasma synoviae* (75), *Vibrio cholerae* (76), *Klebsiella pneumoniae* (77), *Toxoplasma gondii* (78), SARS-CoV-2 (79), etc.

This study sought to design a multi-epitope vaccine against MAP that has the potential to provide cross-protection among various animal species. We conducted a pan-genome analysis of whole-genome sequences of MAP strains from different hosts to identify conserved genes. Furthermore, we performed an antigenicity assessment of proteins located in the ectoplasmic, extracellular, and outer membrane regions from the core proteome of diverse MAP strains. Notably, extracellular, outer membrane, and periplasmic proteins often present immunogenic antigens, making them readily accessible to immune cells (80). Among these five extracellular proteins (group 6283, LpqH, group 7515, group 7334, FbpA) with high antigenicity and were chosen for inclusion in the vaccine construction. While these proteins have been reported in *Mycobacterium tuberculosis* (81, 82), they have not been previously studied in MAP.

Developing a multi-subunit vaccine necessitates the identification of MHC-I and MHC-II restricted epitopes which could be recognized by diverse T cell receptor (TCR) clones. Conversely, B cell epitopes stimulate robust humoral immune responses against specific antigens, broadening the scope of viral treatment. This process involves the selection of appropriate adjuvants and linkers to enhance immunogenicity (33, 83, 84). As such, antigenic, non-toxic, and non-allergenic CTL, HTL, and B-cell epitopes were predicted from all five proteins, forming the basis for constructing the vaccine.

We employed the AAY and GPGPG linkers to enhance improve the presentation of epitopes, significantly bolstering vaccine immunogenicity (85, 86). The AAY linker was strategically positioned to interconnect CTL (MHC I) epitopes, aiding their effective presentation. This linker acted as a cleavage site within the cytoplasm, which facilitated processing by proteasomal and lysosomal degradation pathways. Consequently, the C-terminal end of the epitopes bound efficiently to the TAP protein complex, which transported them to the endoplasmic reticulum (ER). There, the epitopes paired effectively with MHC I molecules, amplifying the CTL response. Additionally, the GPGPG linker reduced conformational flexibility, enhancing antigen presentation and immune processing. To optimize bioactivity and expression, the adjuvant and vaccine sequences were fused using the EAAAK linker (87), an amino acid sequence with inherent adjuvanticity, which can effectively engage TLR4 on dendritic cells (DCs), thereby stimulating naive T cells, polarizing CD4^+^ and CD8^+^ T cells to secrete IFN-γ, inducing T cell-mediated cytotoxicity, and increasing the pool of effector memory cells (88, 89).

*Mycobacterium avium* subsp. *paratuberculosis* (MAP) employs several immune evasion strategies to persist within the host. MAP can prevent the proper activation of the innate immune system by inhibiting the production of pro-inflammatory cytokines and avoiding phagolysosomal fusion, allowing it to persist within macrophages (90–92). Additionally, MAP interferes with antigen processing and presentation, downregulating major histocompatibility complex (MHC) class II molecules and modulating T cell activation. These immune evasion strategies enable MAP to survive and replicate within the host, even in the face of a mounting immune response (93).

To test if the vaccine candidate could counter these evasion mechanisms, we integrated findings from molecular docking, molecular dynamics (MD) simulations, and immune simulation to provide a comprehensive evaluation of the vaccine’s immunogenicity and its potential to thwart MAP’s immune suppression. The molecular docking and MD simulations demonstrated a stable interaction between the vaccine and TLR4, indicating effective receptor engagement and activation of downstream signaling pathways. These results align with the immune simulation, which showed a significant upregulation in antibody titers, enhanced activation of B cells, helper T cells, cytotoxic T cells, antigen-presenting cells (APCs), as well as elevated cytokine levels, indicating a robust immune response. Together, these findings suggest that the vaccine effectively stimulates TLR4-mediated immune activation, countering MAP’s immune evasion mechanisms, such as impaired antigen presentation and cytokine suppression. We emphasize the need for future experimental validation to corroborate these predictions and evaluate the vaccine’s efficacy *in vivo*.

Besides, molecular dynamics (MD) simulation demonstrated the stability of the complexes formed. The predicted expression profile of the vaccine in *E. coli* indicates a high potential for efficient expression based on in-silico cloning results. The codon adaptation index and GC content suggest that the vaccine will be favorably expressed in the *E.coli*, which further indicates the vaccine candidate’s feasibility for future production and application.

## 5 Conclusion

Utilizing pan-genomes and reverse vaccinology approaches, this study designed a multi-epitope vaccine against MAP. Bioinformatics analyses confirmed its stable 3D conformation and ability to evoke potent immune responses. Furthermore, the vaccine candidate is simulated to strongly bind with TLR2 and TLR4 receptors and activate downstream pathways, indicating a potential to overcome MAP’s immune evasion. In summary, this multi-epitope vaccine emerges as a promising vaccine candidate against MAP infection. To further validate the vaccine candidate’s effectiveness, more *in vitro* and *in vivo* experiments would be conducted in our future plan.

## Data Availability

The original contributions presented in the study are included in the article/**Supplementary material**. Further inquiries can be directed to the corresponding authors.
